# Can Ethylene Inhibitors Enhance the Success of Olive Somatic Embryogenesis?

**DOI:** 10.3390/plants11020168

**Published:** 2022-01-09

**Authors:** Muhammad Ajmal Bashir, Cristian Silvestri, Amelia Salimonti, Eddo Rugini, Valerio Cristofori, Samanta Zelasco

**Affiliations:** 1 Council for Agricultural Research and Economics (CREA), Research Centre for Olive, Citrus and Tree Fruit, C/da Li Rocchi Vermicelli, 87036 Rende, Italy; muhammadajmal@unitus.it (M.A.B.); amelia.salimonti@crea.gov.it (A.S.); samanta.zelasco@crea.gov.it (S.Z.); 2 Department of Agriculture and Forest Sciences, University of Tuscia, Via San Camillo De Lellis s.n.c., 01100 Viterbo, Italy; rugini@unitus.it (E.R.); valerio75@unitus.it (V.C.)

**Keywords:** cobalt chloride, de novo organogenesis, *Olea europaea* L., salicylic acid, silver nitrate

## Abstract

An efficient in vitro morphogenesis, specifically through somatic embryogenesis, is considered to be a crucial step for the application of modern biotechnological tools for genetic improvement in olive (*Olea europaea* L.). The effects of different ethylene inhibitors, i.e., cobalt chloride (CoCl_2_), salicylic acid (SA), and silver nitrate (AgNO_3_), were reported in the cyclic somatic embryogenesis of olive. Embryogenic callus derived from the olive immature zygotic embryos of the cultivar Leccino, was transferred to the expression ECO medium, supplemented with the ethylene inhibitors at 20 and 40 µM concentrations. Among these, the maximum number of somatic embryos (18.6) was obtained in media containing silver nitrate (40 µM), followed by cobalt chloride (12.2 somatic embryos @ 40 µM) and salicylic acid (40 µM), which produced 8.5 somatic embryos. These compounds interfered on callus traits: white friable embryogenic calli were formed in a medium supplemented with 40 µM cobalt chloride and salicylic acid; in addition, a yellow-compact embryogenic callus appeared at 20 µM of all the tested ethylene inhibitors. The resulting stimulatory action of silver nitrate among all the tested ethylene inhibitors on somatic embryogenesis, clearly demonstrates that our approach can efficiently contribute to the improvement of the current SE protocols for olive.

## 1. Introduction

Olive (*Olea europaea* L.) plants belong to the family *Oleaceae*, and are one of the most popular species of the genus *Olea*, which is commonly grown in the Mediterranean region and used for food purposes [1]. Furthermore, more than 750 million olive trees are cultivated worldwide. Among the more than one thousand known varieties, of which there are about 600 in Italy, very few are suitable for modern cultivation systems, and the development of novel cultivars is often hampered by the most commonly used breeding techniques that are time-consuming [2]. Although olive, in general, is difficult to be manipulated, in vitro, many cultivars have been established in vitro and micro propagated [3]. Moreover, in vitro culture has always been an excellent tool to support the various laboratory techniques used for genetic, microbiological, physiological, and biochemical studies [4], including biotechnological approaches.

In vitro morphogenesis through somatic embryogenesis is considered as the fundamental step for the application of different biotechnological tools for unconventional breeding in many fruit species, including olives [1]. Previously, somatic embryogenesis has been successfully achieved from zygotic embryos [5,6], radicle and cotyledon segments derived from mature embryos [7,8,9,10]. Furthermore, somatic embryogenesis has also been achieved from mature tissues [1,11,12,13,14,15]. The induction and regeneration of somatic embryos are highly sensitive to culture conditions, such as the medium composition, physical environment of the culture, and the genotype and explant source, especially in olives [1,2,15,16].

There are various factors that influence the somatic embryogenesis response in different plant species. Among them, ethylene is known to inhibit in vitro morphogenetic responses in a genotype-specific manner [17]. Ethylene is recognized as a ubiquitous plant hormone, which has a wide variety of effects on the growth and development of intact plants [18]. It is one of the compounds produced during in vitro culture vessels up to a physiological threshold [19]. The involvement of ethylene in plant tissue growth and differentiation has been widely investigated [20]. Previous studies have demonstrated that ethylene could influence in vitro morphogenetic response in plants [21,22,23]. Therefore, regulation of ethylene perception or ethylene biosynthesis seems to be a promising approach for increasing the efficiency of tissue culture protocols in plant systems. Among these ethylene inhibitors, silver nitrate has been known to inhibit ethylene action [24], and cobaltous ions inhibit ethylene biosynthesis [25], whereas salicylic acid has also been proven to be a potent inhibitor of ethylene biosynthesis [26].

Plant regeneration systems, such as organogenesis and somatic embryogenesis (SE), are eminent micropropagation processes that are based on plant cell totipotency. In organogenesis, plant organs, such as shoots, roots, and even flowers, can be formed from cultured explants. However, for micropropagation purposes, the most interesting factor is de novo shoot meristem formation followed by shoot growth and rooting [27], whereas SE is a more complex developmental pathway, by which the bipolar structures identical to zygotic embryos are developed from the somatic cells through a complex dedifferentiation process, followed by totipotency acquisition and the formation of somatic embryos [28,29]. Since the production of ethylene is directly involved in explant browning and plant morphogenesis under in vivo and in vitro conditions, the inclusion of the compounds that inhibit ethylene biosynthesis are a good alternative to modulate morphogenesis in plant cell and tissue culture. Although different ethylene inhibitors are reported to promote in vitro shoot organogenesis in various economically important plant species [30], the mechanism of their stimulatory effect in olive has not yet been exercised. In the present study, we establish a more efficient and reliable protocol for the induction of somatic embryogenesis in olives, by studying the involvement of different ethylene inhibitors in the culture media.

## 2. Results and Discussion

**Figure 1:**
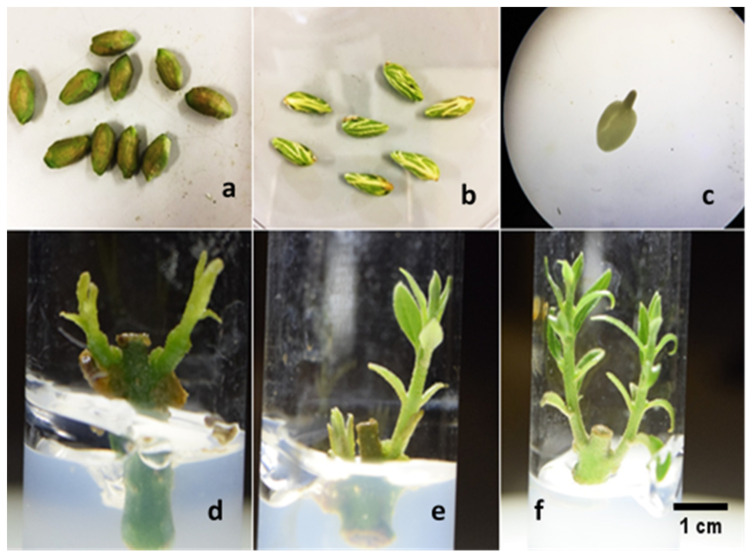
Lignified endocarp containing immature seeds (**a**), seeds released from the sclerified endocarp (**b**), naked embryos after endocarp, and seed coat and endosperm removed (**c**). Nodal explant producing hyperhydrated shoots, usually unable to convert into normal ones (**d**), normal shoots originated from the buds of the nodal explants of the cultivars F7P3 (**e**), and CS-3T (**f**). (Scale bar = 1 cm).

The control of biotic contamination is one of the major concerns in the in vitro establishment of olive material. However, the establishment of callus culture starting from the immature zygotic embryos was very easy due to the absence of endogenous contaminants inside the seeds, and because the olive stones, more susceptible to contaminants, can be strongly surface disinfected (Figure 1a), without compromising the viability of the well protected embryos (Table 1) (Figure 1b,c).

For the in vitro establishment of the cultivars F7P3 and CS-3T, the disinfection procedure used was very efficient, with more than 40% of aseptic explants, with greater success in the “F7P3”, as confirmed by the statistical analysis (Table 1). The differences observed among the cultivars are in line with the literature, and confirm that olive micropropagation, including the phase of in vitro establishment, are genotype dependent [2]. Subsequently, the buds of the aseptic nodal explants were able to convert into normal shoots in both cultivars, CS-3T and F7P3 (78.4 and 69.8%, respectively) (Figure 1e,f). These differences observed between the cultivars are in line with the literature, and confirm that olive micropropagation, including the phase of in vitro establishment, are genotype dependent [2]; only a few shoots were hyperhydrated (Figure 1d), which rapidly became necrotic.

**Figure 2:**
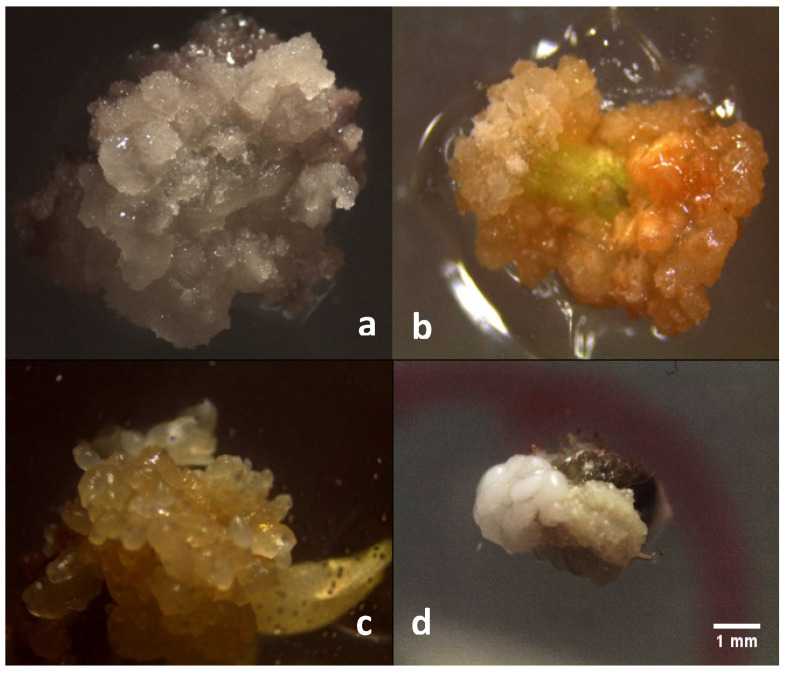
White callus derived from the zygotic embryos of open-pollinated cv. Leccino (**a**) and the yellowish callus derived from the shoot apex of the cultivar CS-3T (**b**). Embryo-like structures in CS-3T (**c**) and the aggregation of well-formed embryos on the callus surface of cultivar F7P3 (**d**). (Scale bar = 1 mm).

In the induction medium, the callus formation from both the immature zygotic embryos and from the shoot apex (leaf primordia) of the cultivars F7P3 and CS-3T, has been observed after 3–4 weeks of culture. The putative embryogenic calli derived from the immature zygotic embryos showed a higher callus proliferation rate than those from the cultivars F7P3 and CS-3T (Table 2); in particular, the calli derived from the embryos were white and friable (Figure 2a), while most of the calli derived from cultivars F7P3 and CS-3T appeared heterogeneous, yellowish and compact (Table 2 and Figure 2b). Surprisingly, by the end of the 4th week of culture on the induction medium, some embryo-like structures (Figure 2c) or embryos (Figure 2d) appeared on the three genotypes.

**Figure 3:**
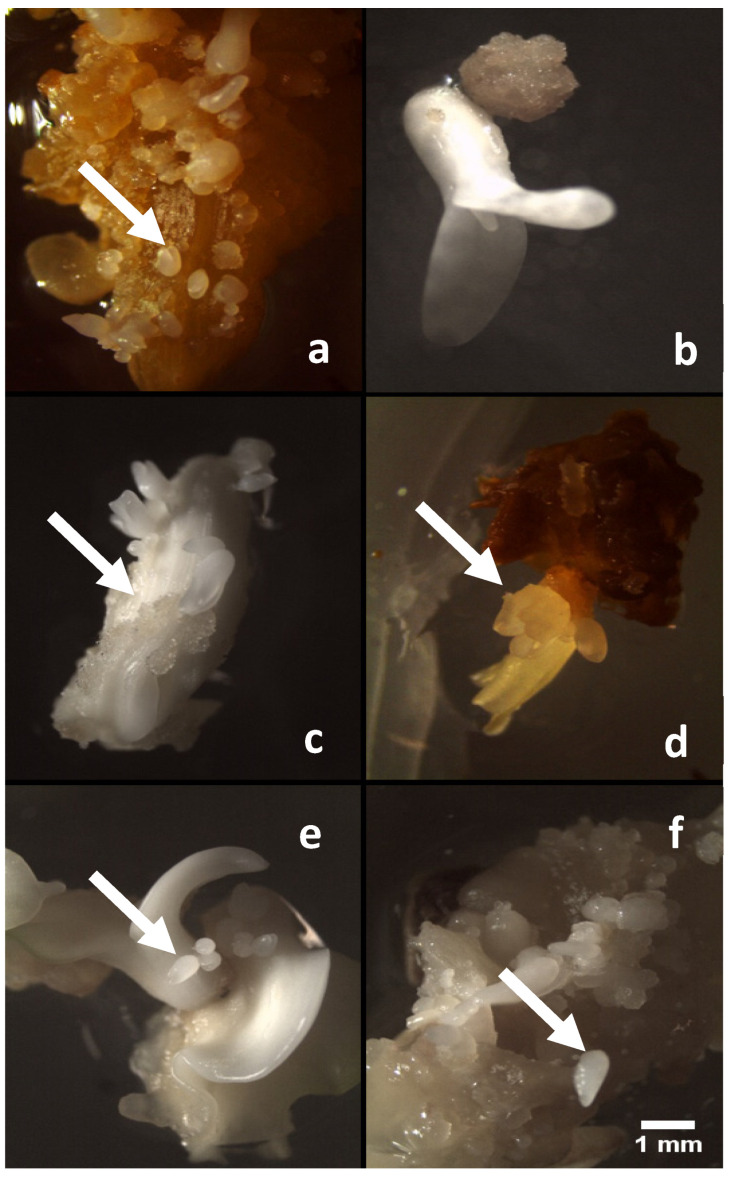
Effect of the ethylene inhibitors on somatic embryogenesis from callus derived from zygotic embryos at different concentrations of AgNO_3_ 20 µM (**a**), salicylic acid 20 µM (**b**), CoCl_2_ 20 µM (**c**), AgNO_3_ 40 µM (**d**), salicylic acid 40 µM (**e**), and CoCl2 40 µM (**f**), after four weeks in culture. (Scale bar = 1 mm). White arrows show the well-formed embryos.

On the expression medium, callus growth was observed in all the media containing different combinations of ethylene inhibitors and in the control medium (lacking ethylene inhibitors). In medium supplemented with 40 µM of AgNO_3_, after the third week of culture, the callus showed a higher percentage of embryogenic callus formation (68.71%), followed by 40 µM of SA treatment with 60.4% of embryogenic callus. As expected, the lowest percentage of embryogenic callus (15.3%) was observed in medium without any ethylene inhibitors (control) and, furthermore, failed to turn into somatic embryos in most cases, allowing to the recovery of only a few normal embryos (Figure 3d). Statistical analysis showed that the rates of callogenesis were not significantly different among the media containing the ethylene inhibitors AgNO_3_ and SA at concentrations of 20 µM, compared to CoCl_2_, which produced a statistically lower percentage at 20 µM. Silver nitrate (AgNO_3_) tested at all concentrations (20 and 40 µM) induced markedly higher numbers (9.2 and 18.6, respectively) of the somatic embryos per explant. However, the somatic embryos obtained from 20 µM of AgNO_3_ turned a deep brown color and could not convert into complete somatic embryos. Among all these ethylene inhibitors, the maximum number of somatic embryos was produced in media containing 40 µM (AgNO_3_) (Figure 3d), followed by 40 µM (CoCl_2_), 20 µM (AgNO_3_), and then SA (40 µM) (Figure 3a,e,f). The embryogenic calli growing on 40 µM of cobalt chloride (CoCl_2_) and salicylic acid produced compact somatic embryo-like structures, but they were unable to develop into a complete cotyledonary structure. Concerning the appearance of callus, friable calli were produced on medium supplemented with 20 µM of cobalt chloride (Figure 3c), and off-white-to-yellow colored calli were observed at 20 µM of SA. The globular embryos obtained from the different treatments have been able to convert into plants without any difference attributable to the type of ethylene inhibitor used.

The results presented in this experiment confirm that the use of ethylene inhibitors in culture media can enhance the ability of olive tissue cultures to produce a higher number of somatic embryos per explant. The highest number of somatic embryos was achieved on media supplemented with 40 µM of AgNO_3_. This result agrees with the previously reported findings demonstrating the stimulative role of AgNO_3_ on shoot organogenesis in many plant species, such as banana [30], Coffea [31], strawberry [32], sweet potato [33], sesame [34], tomato [35], and turmeric [36]. For CoCl_2_ and SA treatments, a lower number of somatic embryos were achieved, compared to the AgNO_3_ treatment. It is well known that AgNO_3_ is a potent inhibitor of ethylene action [37], whereas CoCl_2_ and SA are known to inhibit the enzymes aminocyclopropane-1-carboxylic acid (ACC) synthase and ACC oxidase involved in ethylene biosynthesis [38,39].

There are several reports in literature that clearly show that ethylene influences callus growth, shoot regeneration, and somatic embryogenesis in other plant species [40,41], but not in olive. However, in the present study, the treatment with silver nitrate significantly increased the SE production. Therefore, it can be suggested that silver nitrate is an ethylene action inhibitor, which affects somatic embryogenesis by increasing or decreasing the in vitro response of explants, depending on the species [42,43].

The precise mechanism of ethylene inhibitor action on plants is still uncertain. However, few existing evidences suggest their interference in the ethylene perception mechanism. Recently, AgNO_3_ has been employed in plant tissue culture studies for inhibiting ethylene action due to its water solubility and lack of phytotoxicity at certain concentrations [44]. To summarize, the findings of this study demonstrate that ethylene inhibitors, particularly AgNO_3_ and to a lesser extent CoCl_2_ and SA, enhanced the somatic embryogenic ability in olive explants (Table 3). Therefore, the effect of silver nitrate on somatic embryogenesis can be carefully evaluated for each species. In our study, the resulting stimulatory action of silver nitrate among other ethylene inhibitors tested on somatic embryogenesis, clearly demonstrated its effective contribution to improve the somatic embryogenesis protocols for olive.

## 3. Materials and Methods

### 3.1. Plant Material

Immature zygotic embryos were taken from adult and healthy, and open-pollinated olive trees in cv. Leccino, sixty days after full bloom. The pulp of the immature fruits has been eliminated (Figure 1a) and the endocarp (stone) was washed in running tap water, then submerged in a solution of commercial bleach 20% *v/v* and a few drops of Tween-80 for 20 min. Finally, the olive stones were washed again with sterilized autoclaved water and dried in a laminar flow hood under aseptic conditions. The disinfected stones were opened by using a nutcracker, and the seeds were removed (Figure 1b) and used immediately or stored in Petri dishes in a refrigerator (10 °C) for a week to extract the embryos using a knife. Immature zygotic embryos (Figure 1c) were cultured in half-strength MS medium (including vitamins) supplemented with 0.44 mg L^−1^ BAP. Callus produced after 4 weeks was then used as the plant material for our study.

Regarding the adult material, two cultivars have been used. The clone F7P3 an CS-3T have been established in vitro from potted plants grown in a greenhouse. Briefly, the distal portion of the twigs of the potted plants were cut and the plants were sprayed with fungicides, 7 days and 24 h prior to harvesting the explants to be used for the in vitro establishment. The nodal explants were rinsed in running tap water, then immersed for 30 s in ethanol 70% (*v/v*), and then soaked for 15–30 min in an aqueous solution of ascorbic acid 250 mg L^−1^ and PPM^®^ (Plant Preservative Mixture) 0.1% (*v/v*). Decontamination was performed with a solution of commercial bleach 20% and a few drops of Tween-80 for 20 min. The explants were rinsed three times in sterile deionized water and placed in 15 mL tubes containing medium consisting of OM medium, including vitamins [33] supplemented with mannitol 3.6%, L-Glutamine 2.2 g L^−1^, zeatin (4.56 µM), and GA_3_ 1.44 µM (added filter sterilized after autoclaving). The aseptic neo-formed shoots have been dissected and transferred to a new proliferation medium [3].

### 3.2. Somatic Embryogenesis Induction

Putative embryogenic lines were initiated from the radicles and cotyledons of immature zygotic embryos (the immature zygotic embryos were obtained with the procedure explained in Section 3.1), and cultured for three weeks on half-strength MS medium supplemented with Thidiazuron (TDZ) 22.7 µM, 6-benzylaminopurine (BAP) 4.44 µM, and 2% of sucrose. (All the chemicals used in this study were procured from Duchefa Biochemie, Haarlem, Netherlands)

To induce embryogenic calli from the adult material of the studied cultivars, the shoot apex with emerging leaf primordia with or without the first pair of developing leaves in mature wild olive, as suggested by Narvaez et al. [15], have been cut from 21-day-old micro-shoots, and the protocol adopted by [12] has been followed. Briefly, the above-described explants have been cultured in liquid medium consisting of half-strength MS medium, full strength of MS vitamins, 100 mg L^−1^ of myo-inositol, 30 µM of TDZ, and 0.54 µM of NAA, and maintained in a 50 mL Falcon tube for 4 days in dark conditions at 24 ± 1 °C, on an orbital shaker at 100 rpm. The amount of callus formation has been estimated by an arbitrary scale (0 no callus, 1: <40%, 2: 40–80%, and 3: 80–100%) for the visual criteria (as recommended by [15]).

### 3.3. Expression Phase and Maturation

After 4 weeks, calli derived from the immature zygotic embryos and from adult material were transferred to a modified expression ECO medium [1] containing 1/4 macro-OM; 1/4 micro-MS; ½ NN vitamins [45]; 1g L^−1^ casein hydrolysate; 0.55g L^−1^ L-glutamine; and 2% sucrose; supplemented with 0.4 µM of benzylaminopurine (BAP), 0.49 of µM 6-(γ, γ-dimethylallylamino) purine (2iP), 0.25 µM indole-3-butyric acid (IBA), and cefotaxime (200mg L^−1^). The medium was supplemented with three different ethylene inhibitors: silver nitrate (AgNO_3_), salicylic acid (SA), and cobalt chloride (CoCl_2_) at two concentrations (20 µM and 40 µM). a survey on somatic embryogenesis has been carried out after four weeks in culture. The globular somatic embryos derived from the embryogenic calli of the zygotic embryos from Leccino have been subjected to maturation and conversion, as described by [9]. Briefly, embryos were placed in multiwells with an ECO medium supplemented with 1g L^−1^ activated charcoal; after 6 weeks the mature embryos were transferred to half-strength OM medium containing mannitol (3.6%).

### 3.4. Culture Conditions

All culture media were adjusted to pH 5.8 with 1M NaOH or HCl, before adding the gelling agent. Both the induction and expression media were solidified with gelrite at 3 g L^−1^, while other maintenance and regeneration media were solidified with plant agar at 5.8 g L^−1^. All media were autoclaved at 121 °C for 20 min. The induction and expression cultures were incubated in the dark with controlled conditions in a growth chamber at 24 ± 1 °C, while the proliferation of shoots was routinely carried out under a 16 h photoperiod, 40 µmol m^−2^s^−2^ PPFD (white LED lights), and at a temperature of 23 ± 1 °C.

### 3.5. Statistical Analysis

All data was processed by XLSTAT integrated into Microsoft Excel. All the parameters were comprised of three replicates each, and then subjected to analysis of variance (ANOVA) and *t*-test. A Duncan post hoc multiple range test was used for the mean separation and to provide homogeneous groups for the means (at *p* ≤ 0.05).

## 4. Conclusions

In this study, an efficient and rapid protocol was developed for a more efficient somatic embryogenesis by using ethylene inhibitors, which has paved a path to overcome the double regeneration technique that has been previously adopted for mature tissues of olive cultivars [11]. In addition, this technique is still essential to maintain the morphogenetic callus for many subcultures in several woody species, including olive [6]. Silver nitrate has a stimulatory effect on the somatic embryogenesis of olives, depending on its concentration; AgNO_3_ at 40 µM was the best treatment, producing the highest frequency of somatic embryogenesis in olives, compared to other ethylene inhibitors tested, such as salicylic acid and cobalt chloride. The AgNO_3_ supposedly inhibits ethylene action by competing with ethylene for the binding sites, and the silver ion can replace the cofactor single copper ion (Cu) present in the ethylene-binding site of the ethylene receptor and lock it to continuously suppress the ethylene response [46]. The positive role of silver nitrate in somatic embryogenesis can contribute to unraveling the recalcitrant nature of the olive species. In future research, it would be advantageous to test the effectiveness of silver nitrate in combination with other ethylene inhibitors for the efficient somatic embryogenesis in olives, particularly cobalt chloride, which also appears as a very promising molecule to be tested in higher concentrations.

## Figures and Tables

**Table 1:** The contamination rates of the different explant sources (immature embryos of cv. Leccino and uni-nodal explants of the cultivars CS-3T and F7P3) and the bud development from the aseptic nodal explants of both cultivars. Data have been shown as the mean ± standard deviation. The mean denoted by different letters are significantly different (Duncan’s test, *p* < 0.05).

Explant Source	Contamination Rate (%)	Explants Forming Shoots (%)
Zygotic Embryo of Leccino	2.1 ± 0.7	-
Nodes of CS-3T	56.4 ± 3.8 a	78.4 ± 8.8
Nodes of F7P3	33.2 ± 2.9 b	68.9 ± 10.2

**Table 2:** The callus formation of the different explant sources of the immature embryos of cv. Leccino, and the leaflets of growing shoots from the varieties CS-3T and F7P3, after 4 weeks on the induction medium. Data are reported as the mean ± standard deviation. The mean denoted by different letters are significantly different (Duncan’s test, *p* < 0.05). The amount of callus formation has been estimated by an arbitrary scale (0 no callus, 1: < 40%, 2: 40–80%, and 3: 80–100%) on visual criteria, as recommended by [15].

Explant Source	Explant Forming Callus (%)	Amount of Callus	Callus Traits
Zygotic Embryo Leccino	100	2.8 ± 0.3 a	White and friable
Variety CS-3T	90 ± 5	2.2 ± 0.3 b	White/yellowish compact
Variety F7P3	95 ± 3	1.9 ± 0.2 b	Yellowish and compact/friable

**Table 3:** The effect of ethylene inhibitors on the percentage of embryogenic callus formation and the number of somatic embryos recovered. The data are reported as the mean ± standard deviation. The mean denoted by different letters are significantly different (Duncan’s test, *p* < 0.05).

Ethylene Inhibitors	Embryogenic Callus (%)	Number of Somatic Embryos per Callus
Control	15.3 ± 2.1 cd	3.8 ± 0.3 d
AgNO_3_ 20 µM	23.8 ± 4.2 c	9.2 ± 0.3 bc
AgNO_3_ 40 µM	68.7 ± 8.1 a	18.6 ± 0.2 a
SA 20 µM ^y^	26.0 ± 4.5 bc	6.0 ± 0.3 cd
SA 40 µM	60.4 ± 4.1 b	8.5 ± 0.2 bc
CoCl_2_ 20 µM	12.2 ± 1.5 d	7.3 ± 0.3 c
CoCl_2_ 40 µM	28.1 ± 4.0 bc	12.2 ± 0.2 b

^y^ SA (salicylic acid).
